# A Novel Oxidative Phosphorylation-Associated Gene Signature for Prognosis Prediction in Patients with Hepatocellular Carcinoma

**DOI:** 10.1155/2022/3594901

**Published:** 2022-09-05

**Authors:** Weigang Chen, Zelong Yang, Yong Chen

**Affiliations:** ^1^Department of Hepatobiliary Surgery, Xijing Hospital, Air Force Medical University, Xi'an 710032, China; ^2^Department of General Surgery, Air Force Hospital of Western Theater Command, Chengdu 610021, China

## Abstract

Hepatocellular carcinoma (HCC) is a common type of malignant tumor with high morbidity and mortality. The oxidative phosphorylation (OXPHOS) metabolic pathway produces adenosine triphosphate (ATP) by delivering electrons to transmembrane protein complexes in the mitochondria. This research was dedicated to identifying an OXPHOS-associated signature for the assessment of prognosis of HCC patients. A total of 371 HCC patients from the Cancer Genome Atlas (TCGA) and 231 HCC patients from the International Cancer Genome Consortium (ICGC) with RNA expression data and clinical data were employed as construction and validation cohorts, respectively. The least absolute shrinkage and selection operator (LASSO) Cox regression was applied to establish a multigene signature in the TCGA cohort, and the ICGC cohort was used for validation. The prognostic value of the risk signature was evaluated using univariate and multivariate Cox regression, Kaplan–Meier curves, and receiver operating characteristic (ROC) curves. The potential enrichment of biological functions was investigated using Gene Ontology (GO) and Kyoto Encyclopedia of Genes and Genomes (KEGG) pathway analyses. Meanwhile, we analyzed the correlation between the risk score and the tumor microenvironment (TME). A five-gene signature including ATP6V0B, ATP6V1C1, ATP6V1E1, TIMM9, and UQCRH was identified by LASSO Cox regression to classify patients into low- and high-risk groups. ROC curve analysis indicated that the five-gene signature is a prospective prognostic factor in HCC patients. Univariate and multivariate Cox regression analyses demonstrated that the risk score was an independent prognostic factor for overall survival (OS). Functional analysis showed that differentially expressed genes (DEGs) between the low- and high-risk groups were enriched in mitosis and the cell cycle pathway. In addition, the five-gene signature was associated with innate immune cell infiltration, immune subtypes, and tumor stemness. A novel OXPHOS-associated gene signature can be used for prognostic prediction for patients with HCC.

## 1. Introduction

Hepatocellular carcinoma (HCC) is the fourth leading cause of cancer-associated death and ranks sixth in terms of morbidity among all cancers [1]. The most common etiologies of HCC include hepatitis B or C virus infection and alcohol abuse, which can cause liver cirrhosis and chronic inflammation [2]. In addition to comprehensive cancer management, surgery remains the cornerstone of treatment for most patients with HCC [3]. Despite great advances in the treatment of HCC over the past decades, the 5-year survival rate of patients with HCC remains unsatisfactory [1]. Meanwhile, due to the substantial heterogeneity in HCC, it is difficult to evaluate the prognosis of patients with HCC and provide more precise guidance for comprehensive treatment based only on traditional histological classification [4, 5]. Therefore, there is a pressing need to establish a more readily available prognostic evaluation model for HCC.

The oxidative phosphorylation (OXPHOS) metabolic pathway is a fundamental and indispensable process for most cells, which can yield adenosine triphosphate (ATP) by transferring electrons to transmembrane protein complexes in the mitochondrial membrane and subsequently providing energy for the metabolic process [6]. Furthermore, ATP can be released from cells and is involved in different physiological and pathological conditions [7]. Studies have shown that ATP is one of the major components of the tumor microenvironment (TME), and it has been proven to promote or repress certain types of tumor progression [8, 9]. Compared with normal cells, well-oxygenated cancer cells exhibit increased glycolysis, upregulated glucose consumption, and increased lactate production, indicating that OXPHOS is downregulated in cancers [10]. This is true for many cancers. Additionally, OXPHOS downregulation is linked to poor clinical outcomes across several cancer types and is related to the characteristics of invasive or metastatic tumors [11]. However, growing evidence has demonstrated that certain cancers, such as breast cancer, pancreatic ductal adenocarcinoma, melanoma, and lymphomas, are dependent on OXPHOS, which is upregulated in these cancers, and that OXPHOS inhibition is an effective treatment for these cancer subtypes [12, 13]. Furthermore, earlier studies have suggested that increased liver cancer cell migration and proliferation were associated with upregulation of OXPHOS and that decreased OXPHOS can exacerbate cell death and suppress tumor growth in HCC [14–16]. All of these indications suggest that OXPHOS might be involved in the progression of HCC and may represent a potential target for the treatment of HCC subtypes. However, the involvement of OXPHOS in the clinical prognosis and biological features of patients with HCC remains unknown. Thus, it is worthwhile to comprehensively investigate these associations.

In this study, we gathered gene expression and clinical data for HCC patients from the Cancer Genome Atlas (TCGA) and the International Cancer Genome Consortium (ICGC). The HCC patients were divided into two groups by integrating the gene expression and clinical data through LASSO Cox regression. We established a five-gene signature associated with OXPHOS to predict the clinical outcomes of HCC patients and validated it based on the ICGC cohort. In addition, we identified the possible underlying mechanisms using functional enrichment analysis. Then, we analyzed the correlations between the risk score and TME as well as immune status. Ultimately, we identified the associations between drug sensitivity and the five genes of the signature.

## 2. Materials and Methods

### 2.1. Data Collection

The total RNA expression matrix of 371 HCC patients and their corresponding clinicopathological data were obtained from the Cancer Genome Atlas (TCGA) database (http://portal.gdc.cancer.gov/repository). The other RNA expression matrix and clinical data of 231 HCC patients were obtained from the International Cancer Genome Consortium (ICGC) database (http://dcc.icgc.org/projects/LIRI-JP). A total of 200 OXPHOS-associated genes (OAGs) are obtained via the “HALLMARK_OXIDATIVE_PHOSPHORYLATION” gene set in the Molecular Signatures Database (MSigDB) (http://www.gsea-msigdb.org/gsea/msigdb) and are presented in Supplementary Table S1.

### 2.2. Construction and Validation of a Prognostic Oxidative Phosphorylation-Associated Gene Signature

The “limma” R package was used to recognize the differentially expressed genes (DEGs) between tumor and normal samples of HCC patients by analyzing RNA expression data from the TCGA cohort with a false discovery rate (FDR) <0.05 and |log 2 Fold Change (FC)| >1. Univariate Cox analysis was applied to distinguish the OAGs with prognostic value by using the “survival” R package, and the *P* value was adjusted using the Benjamini and Hochberg (BH) correction method. The DEGs and prognosis-related OAGs that overlapped were regarded as candidate prognostic OAGs for model construction and are presented in a Venn diagram using the “Venn” R package. The candidate prognostic OAGs were further used for correlation network analysis to predict the potential interactions among the proteins encoded by these genes, which were presented using the “igraph” and “reshape” R packages. A novel OXPHOS-associated risk signature in HCC was established based on LASSO-penalized Cox regression analysis [17, 18] using the “glmnet” R package. The penalty parameter (*λ*) was verified by tenfold cross-validation following the minimum criteria. The model was established by the following formula: risk score = expression of gene1 × C1 + expression of gene2 × C2 + ⋯+expression of gene *n* × Cn, where Cn is the corresponding coefficient of gene *n*. Based on the above model, the risk scores of HCC tumor samples in the TCGA and ICGC cohorts were obtained. HCC tumor samples in the TCGA and ICGC cohorts were classified into low- and high-risk groups according to the median risk score. Finally, TCGA and ICGC cohorts were used as construction and validation cohorts, respectively. The expression levels of individual genes in the prognostic model between tumor and normal samples and the relationship between the individual genes and overall survival (OS) of HCC patients were analyzed by gene expression profiling interactive analysis (GEPIA) (http://gepia.cancer-pku.cn) using TCGA LIHC tumor data and matched data of normal tissue from TCGA and GTEx [19].

### 2.3. Evaluation of the Gene Signature Accuracy

The distribution of risk groups of the TCGA and ICGC cohorts was presented by t-SNE and PCA analyses using the “Rtsne” and “ggplot2” R packages. The OS analysis between the low- and high-risk groups was compared using the “survival” and “survminer” R packages. The predictive accuracy of the model was verified by the area under the curve (AUC) of the receiver operating characteristic (ROC) curve in the two groups using the “timeROC” R package. The independent prognostic value of the risk score was evaluated through univariate and multivariate Cox regression analyses using the “survival” R package. Parameters with *P* < 0.05 based on the univariate Cox regression analysis were further involved in the multivariate Cox regression analysis.

### 2.4. Functional Enrichment Analysis

Gene Ontology (GO) and Kyoto Encyclopedia of Genes and Genomes (KEGG) enrichment analyses were conducted and visualized by using the “clusterProfiler,” “org.Hs.eg.db” and “ggplot2” R packages to investigate the potential biological functions of the DEGs between the low- and high-risk groups. Biological process (BP), molecular function (MF), and cellular component (CC) were used for GO enrichment analyses. The *P* value was adjusted using the BH method. The infiltration scores of sixteen immune cell subpopulations and the activities of thirteen immune-related pathways between the high- and low-risk groups were computed by single-sample gene set enrichment analysis (ssGSEA) [20] with the “GSVA,” “limma,” and “GSEABase” R packages. The annotated gene set is presented in Supplementary Table S2.

### 2.5. Tumor Microenvironment and Immune Response Analysis

The infiltration levels of immune cells and stromal cells in different tumor samples from the TCGA cohort were determined by immune and stromal scores that were calculated using the estimation of stromal and immune cells in malignant tumor tissues using expression data (ESTIMATE) algorithm [21](https://bioinformatics.mdanderson.org/publicsoftware/estimate/). The correlation between risk score and those scores was determined using the Spearman correlation. Two-way ANOVA was used to determine the correlation between risk score and immune infiltration subtypes. Tumor stem cell features retrieved from the transcriptome and epigenetics data of TCGA tumor tissues were used to quantify stem cell-like features of the tumor. Tumor stemness was measured by the RNA stemness score (RNAss) according to mRNA expression and the DNA stemness score (DNAss) according to the DNA methylation pattern. The Spearman correlation test was used to determine the correlation between tumor stemness and risk score. The results were visualized by the “ggExtra,” “ggplot2,” and “ggpubr” R packages.

### 2.6. Chemotherapy Drug Sensitivity Analysis

The NCI-60 database was obtained through the CellMiner interface (https://discover.nci.nih.gov/cellminer). The association between the signature genes of the prognostic model and drug sensitivity was investigated using Pearson correlation analysis. Analysis of the efficacy of 216 chemotherapy drugs (Supplementary Table S3) approved by the Food and Drug Administration (FDA) was performed. Outcomes were presented using the “limma,” “impute,” “ggplot2,” and “ggpubr” R packages.

### 2.7. Statistical Analysis

The Wilcoxon test was used to compare the DEGs between tumor and normal samples. The Mann–Whitney test was used to compare the ssGSEA scores of immune cells or immunity between the low- and high-risk groups, and the *P* value was adjusted using the BH method. The Kaplan–Meier method was applied to compare the differences in OS across different groups. Univariate and multivariate Cox analyses were used to identify the independent predictors for OS. Spearman or Pearson correlation analysis was used to analyze the association of risk scores or gene expression levels with stemness score, stromal score, immune score, and drug sensitivity. *P* value less than 0.05 demonstrated that the difference was statistically significant. R software (Version 4.1.2) (http://www.r-project.org/) and Perl language (http://www.perl.org) were applied to conduct all analyses.

## 3. Results

The study scheme is presented in Supplementary Figure S1. A total of 365 HCC patients from the TCGA-LIHC cohort and 231 HCC patients from the ICGC (LIRI-JP) cohort were involved. The clinical data of these patients are summarized in Supplementary Table S4.

### 3.1. Screening the Prognostic Genes Associated with OXPHOS in the TCGA Cohort

**Figure 1:**
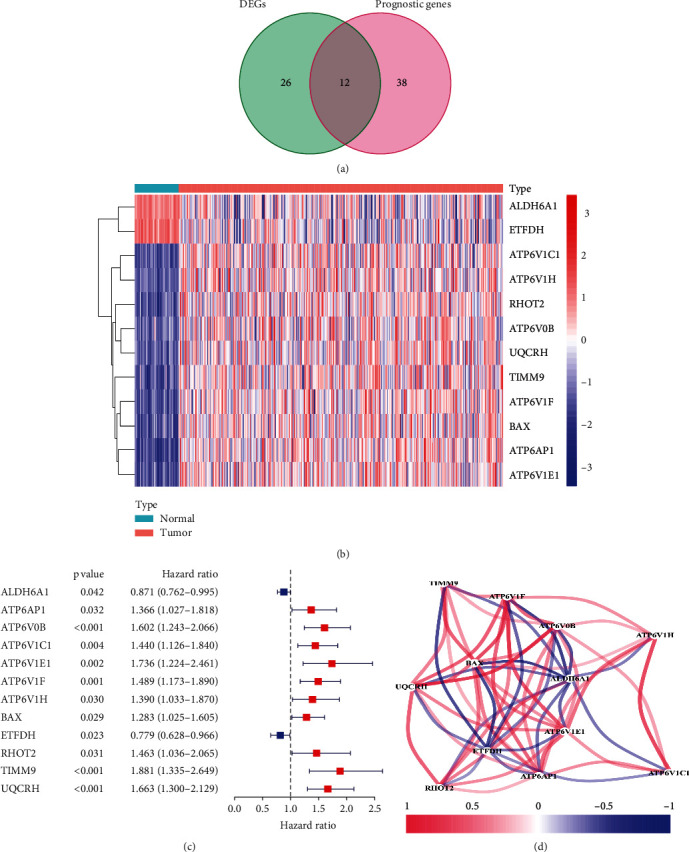
Screening the prognostic genes associated with OXPHOS in the TCGA cohort. (a) Venn diagram of the overlapping DEGs and OAGs with prognostic value. (b) Heatmap of the 12 overlapping genes. (c) The forest plots of associations between the 12 genes and OS of patients were analyzed by univariate Cox regression. (d) The correlation network of the 12 genes.

There were 38 OAGs identified as DEGs between tumor and normal samples within the TCGA cohort. Univariate Cox analysis demonstrated that 50 OAGs were significantly related to the OS of patients with HCC. Subsequent analysis showed that 12 genes were implicated in both DEGs and prognosis-related OAGs (Figure 1(a)). The expression levels of the 12 genes were found to be differentially expressed between tumor and normal samples. The expression of ALDH6A1 and ETFDH was downregulated in tumor samples, and the remaining ten genes were upregulated in tumor samples as compared with normal samples (Figure 1(b)). The hazard ratio (HR) was used to identify the effects of the candidate genes on the prognosis of HCC patients. Univariate Cox analysis demonstrated that ALDH6A1 and ETFDH served protective roles in the prognosis of HCC patients (HR<1), while the other ten genes exhibited adverse effects (HR>1) (Figure 1(c)). The correlation network among the 12 genes was analyzed, and the results revealed that they were correlated with each other. More specifically, ETFDH and ALDH6A1 were negatively correlated with the expression of the other ten candidate genes, while most of the other ten candidate genes were positively correlated with each other. (Figure 1(d)). As a result, the 12 OAGs were regarded as candidate prognostic genes for model construction.

### 3.2. Construction of a Prognostic Model for Patients with HCC in the TCGA Cohort

**Figure 2:**
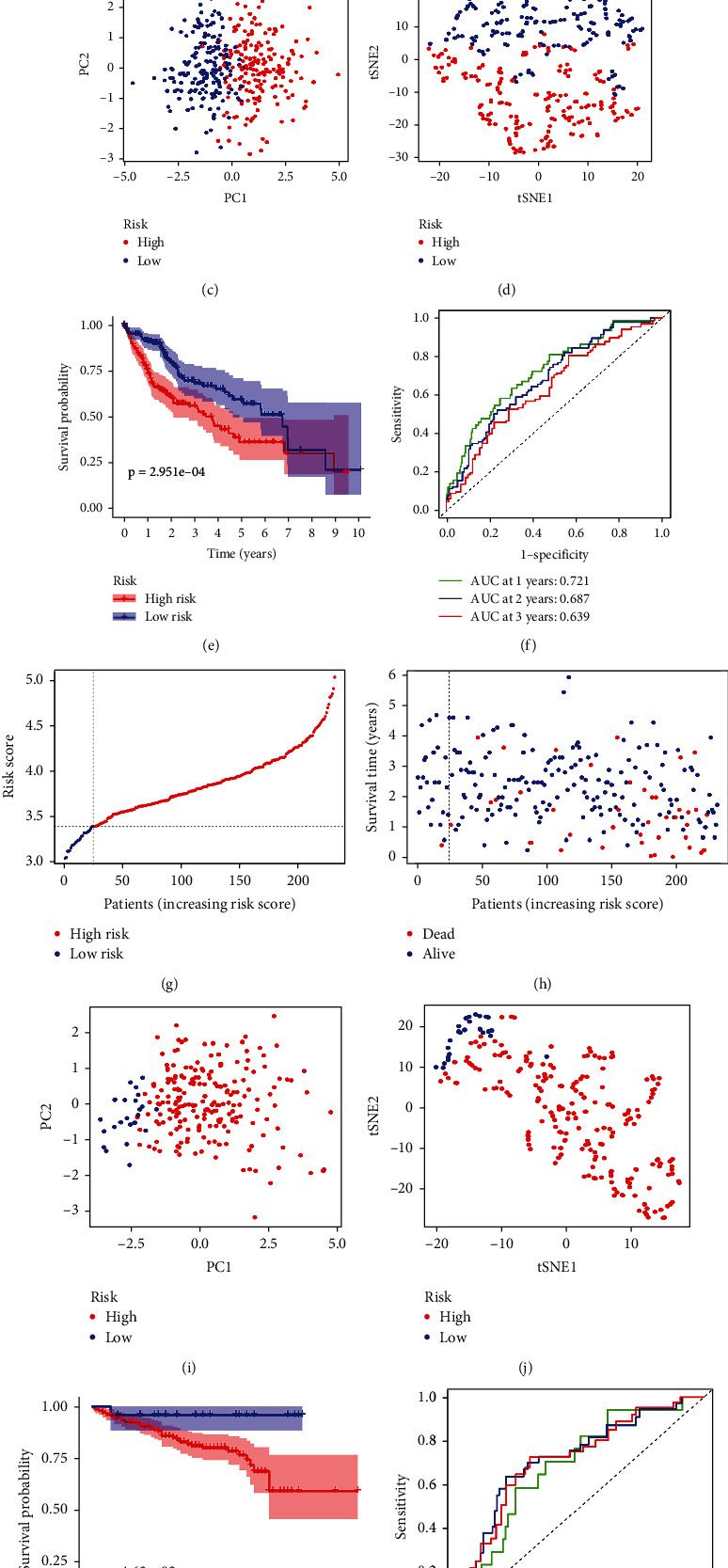
Prognosis analysis of the prognostic model in TCGA and ICGC cohorts. The distribution and median value of the risk score in the TCGA cohort (a) and the ICGC cohort (g). The distributions of survival status and risk scores in the TCGA cohort (b) and the ICGC cohort (h). PCA plot of the TCGA cohort (c) and the ICGC cohort (i). t-SNE analysis of the TCGA cohort (d) and the ICGC cohort (j). The Kaplan–Meier curves of the TCGA cohort (e) and the ICGC cohort (k). The AUC of time-dependent ROC curves used to predict the OS of patients from the TCGA cohort (f) and the ICGC cohort (l).

**Figure 3:**
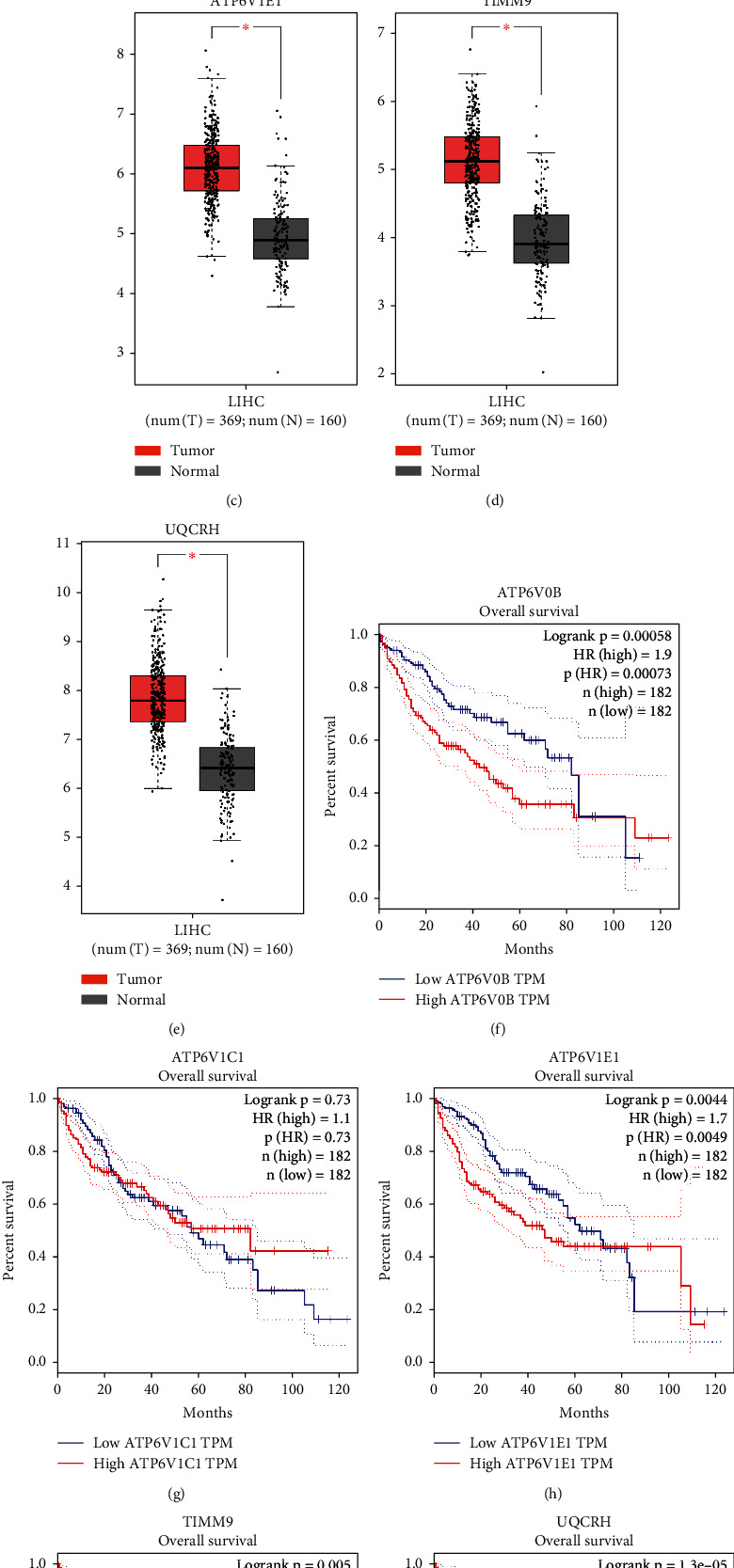
The expression profiles and survival analysis of single genes from the prognostic model. The expression profiles of single genes from the prognostic model differed between the tumor samples and the normal samples (a–e). Kaplan–Meier curves of single genes from the prognostic model (f–j). ^∗^*P* < 0.05.

The expression profiles of the 12 prognostic candidate genes were analyzed by LASSO Cox regression (Supplementary Figure S2). A five-gene prognostic model including ATPase H+ Transporting V0 subunit B (ATP6V0B), ATPase H+ Transporting V1 subunit C1 (ATP6V1C1), ATPase H+ Transporting V1 subunit E1 (ATP6V1E1), translocase of inner mitochondrial membrane 9 (TIMM9), and ubiquinol-cytochrome C reductase hinge (UQCRH) was established according to the optimal value of *λ*. The risk score was calculated using the following formula: risk score = 0.1025 × expression level of ATP6V0B + 0.1635 × expression level of ATPV1C1 + 0.0086 × expression level of ATP6V1E1 + 0.2387 × expression level of TIMM9 + 0.2341 × expression level of UQCRH (Table 1). Patients were classified into two groups according to the median risk scores (Figure 2(a)). Additionally, the plot chart showed that patients in the high-risk group were more prone to die earlier than those in the low-risk group (Figure 2(b)). PCA and t-SNE analysis indicated that patients in the two groups were located in diverse regions of the charts (Figures 2(c) and 2(d)). In addition, the Kaplan–Meier curve demonstrated that the patients in the high-risk group had lower OS than those in the low-risk group (Figure 2(e), *P* < 0.05). Receiver operating characteristic (ROC) curves were used to evaluate the prediction accuracy of the prognostic model. The area under the curve (AUC) was 0.721 at one year, 0.687 at two years, and 0.639 at three years (Figure 2(f)). We then investigated the association between a single gene of the prognostic model and prognosis using GEPIA, which revealed that high expression of these genes was significantly associated with poor OS in patients with HCC, except for ATP6V1C1 (Figures 3(f)–3(j), *P* < 0.05). In addition, the expression levels of the five genes in tumor samples were higher than those in normal samples (Figures 3(a)–3(e), *P* < 0.05).

### 3.3. Validation of the 5-Gene Prognostic Model in the ICGC Cohort

To verify the stability of the 5-gene prognostic model from the TCGA cohort, patients from the ICGC cohort were classified into low- and high-risk groups based on the risk score formula from the TCGA cohort (Figure 2(g)). Survival analysis revealed that patients in the high-risk group were more prone to die earlier (Figure 2(h)) and had a lower OS than those in the low-risk group (Figure 2(k), *P* < 0.05). In line with the TCGA cohort, PCA and t-SNE analyses demonstrated that patients in different groups from the ICGC cohort were spread in different regions of the charts (Figures 2(i) and 2(j)). Additionally, the AUC of the 5-gene signature was 0.690 at one year, 0.726 at two years, and 0.720 at three years (Figure 2(l)).

### 3.4. Independent Prognostic Value of the 5-Gene Prognostic Model

**Figure 4:**
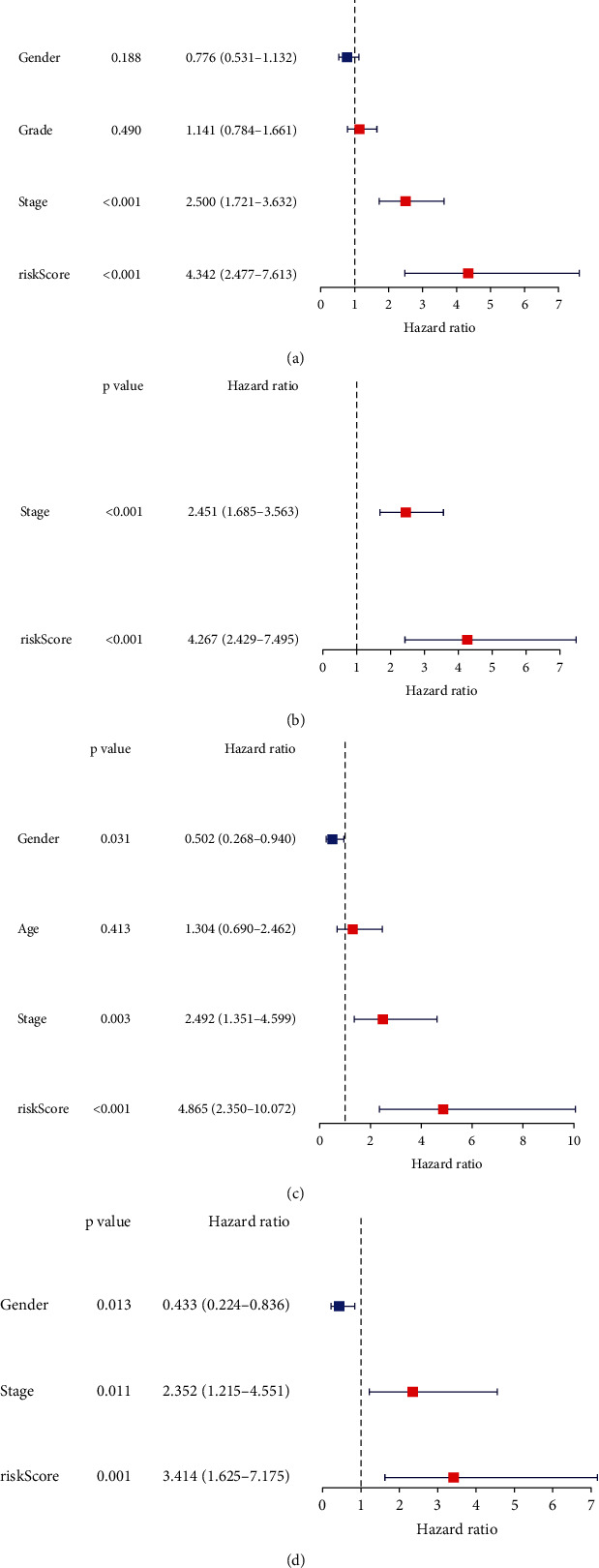
Univariate and multivariate Cox regression analyses concerning OS in the TCGA cohort (a, b) and the ICGC cohort (c, d).

To test whether the risk score of the 5-gene prognostic model was an independent prognostic factor for OS, univariate and multivariate Cox analyses were conducted for both the TCGA and ICGC cohorts. Univariate Cox analysis indicated that the risk score was significantly associated with OS in both cohorts (TCGA cohort: HR = 4.342, 95%CI = 2.477-7.613, *P* < 0.001; ICGC cohort: HR = 4.865, 95%CI = 2.350-10.072, *P* < 0.001; Figures 4(a) and 4(c)). After correcting for other confounding variables, multivariate Cox analysis demonstrated that the risk score remained an independent predictor for OS (TCGA cohort: HR = 4.267, 95%CI = 2.429-7.495, *P* < 0.001; ICGC cohort: HR = 3.414, 95%CI = 1.625-7.175, *P* = 0.001; Figures 4(b) and 4(d)).

### 3.5. Biological Functional Enrichment Analysis in the TCGA and ICGC Cohorts

**Figure 5:**
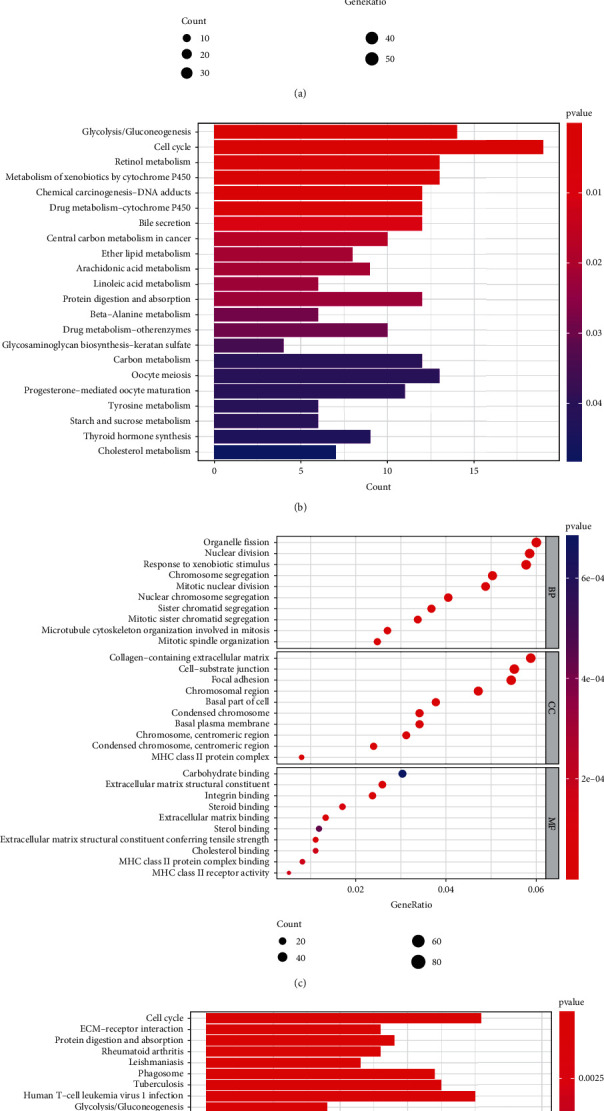
Representative outcomes of GO and KEGG enrichment analyses of the DEGs between the low-risk and high-risk groups. The most significant GO and KEGG enrichment in the TCGA cohort (a, b) and the ICGC cohort (c, d).

GO and KEGG enrichment analyses were conducted to identify the biological functions that correlated with the risk score, and the DEGs between the high- and low-risk groups were utilized to perform the analysis. Based on the GO analysis, the DEGs were enriched in mitotic processes such as mitotic nuclear division, chromosome segregation, and mainly involved in chromosome centromeric region in the TCGA cohort (*P* < 0.05, Figure 5(a)). In accordance with the TCGA cohort, eight mitosis-associated biological processes or components, including organelle fission, nuclear division, chromosome segregation, mitotic nuclear division, sister chromatid segregation, mitotic sister chromatid segregation, chromosome centromeric region, and condensed chromosome centromeric region, were further verified by the ICGC cohort (*P* < 0.05, Figure 5(c)). In addition, according to the KEGG analysis, the cell cycle pathway was significantly enriched in both the TCGA and ICGC cohorts (*P* < 0.05, Figures 5(b) and 5(d)).

### 3.6. Immune Response and Microenvironment Analysis

**Figure 6:**
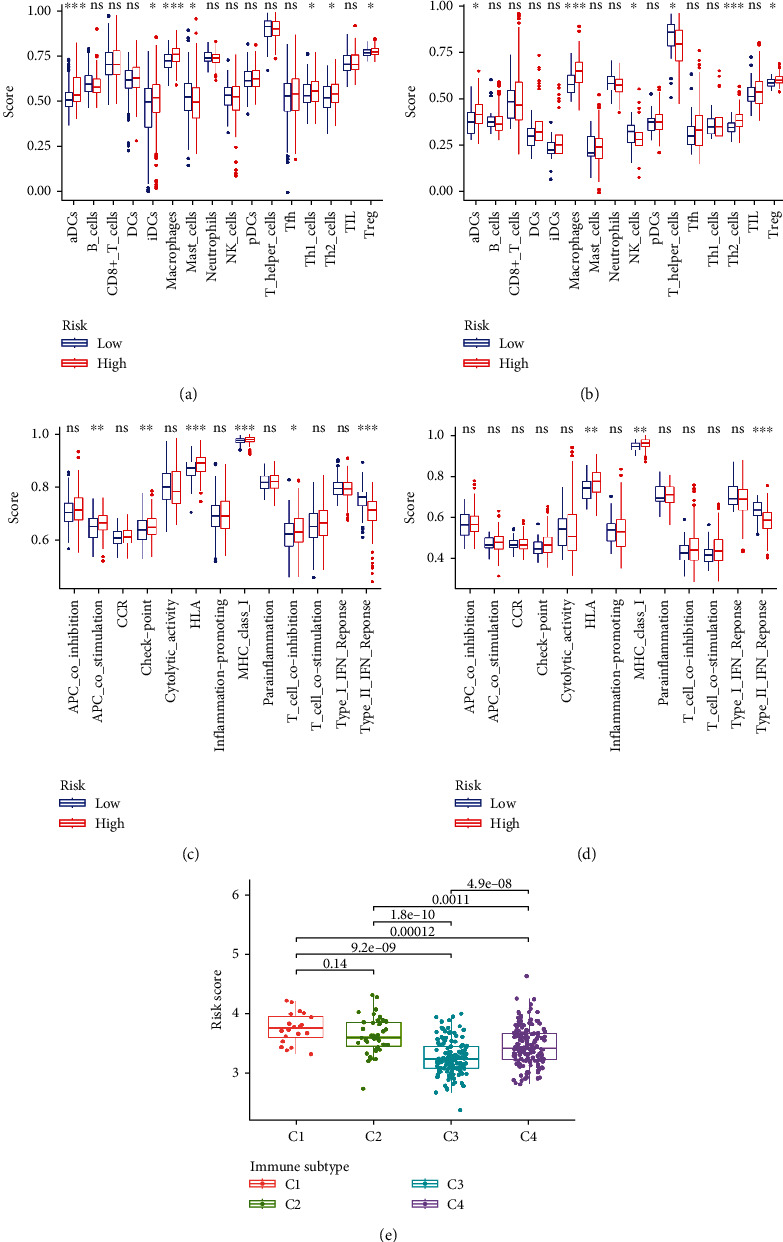
The immune status and infiltration subtypes between different risk groups. The boxplots for scores of sixteen immune cell subpopulations of the low-risk and high-risk groups in the TCGA cohort (a) and the ICGC cohort (b). The boxplots for thirteen immune-associated functions of the low-risk and high-risk groups in the TCGA cohort (c) and the ICGC cohort (d). Correlations between risk score and immune infiltration subtypes (e). The *P* value is presented as: ^∗^*P* < 0.05; ^∗∗^*P* < 0.01; ^∗∗∗^*P* < 0.001; ns indicates “not significant”.

**Figure 7:**
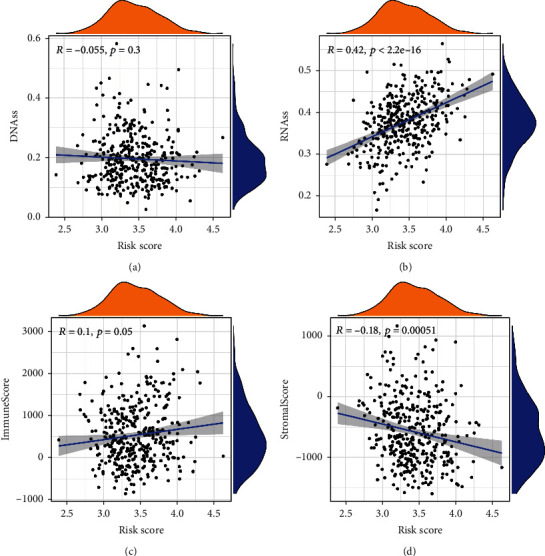
The correlation between the risk score and tumor microenvironment in the TCGA cohort. The correlation between the risk score and DNAss (a), RNAss (b), immune score (c), and stromal score (d).

To explore the relationship between the risk scores and immune response in the TCGA cohort, enrichment scores of different immune cell subpopulations and correlated immune pathways were analyzed using ssGSEA. The results demonstrated that the innate immune cells or antigen presentation process components in the TCGA cohort, consisting of aDCs, iDCs, macrophages, APC co-stimulation, and HLA, were significantly elevated in the high-risk group (*P* < 0.05, Figures 6(a) and 6(c)). In addition, inflammatory-associated responses, including Th1 cells, Th2 cells, Tregs, T cell co-inhibition, and check-points, were elevated in the high-risk group, while the type II IFN response and mast cells were decreased compared to the low-risk group (*P* < 0.05, Figures 6(a) and 6(c)). The comparison results of the ICGC cohort confirmed that aDCs, macrophages, Th2 cells, Tregs, HLA, and MHC class I were elevated in the high-risk group, while NK cells, T helper cells, and the type II IFN response were decreased in the high-risk group compared to the low-risk group (*P* < 0.05, Figures 6(b) and 6(d)). We analyzed the correlation between the risk scores and immune infiltration. Six kinds of immune infiltrate were validated in human tumors, i.e., C1 (wound healing), C2 (IFN-*γ* dominant), C3 (inflammatory), C4 (lymphocyte depleted), C5 (immunologically quiet), and C6 (TGF-*β* dominant). Because no patient was part of the C5 subtype in HCC and just one patient belonged to the C6 subtype, C5 and C6 were not involved in the analysis. We analyzed the immune infiltration of HCC patients in the TCGA cohort and linked it with risk score; the results indicated that high-risk scores were significantly correlated with C1 and low-risk scores were significantly correlated with C3 (Figure 6(e)). Since the immune microenvironment (i.e., immune scores, stromal scores) and tumor stemness (i.e., DNAss and RNAss) are critical regulators of tumor progression, we analyzed the correlation between risk score and the immune microenvironment and tumor stemness. The risk score was positively associated with RNAss but not significantly associated with DNAss (Figures 7(a) and 7(b)). In addition, the results indicated that the risk score was negatively associated with stromal scores but not significantly associated with immune scores (Figures 7(c) and 7(d)).

### 3.7. Associations between Signature Gene Expression and Chemotherapy Drug Sensitivity

**Figure 8:**
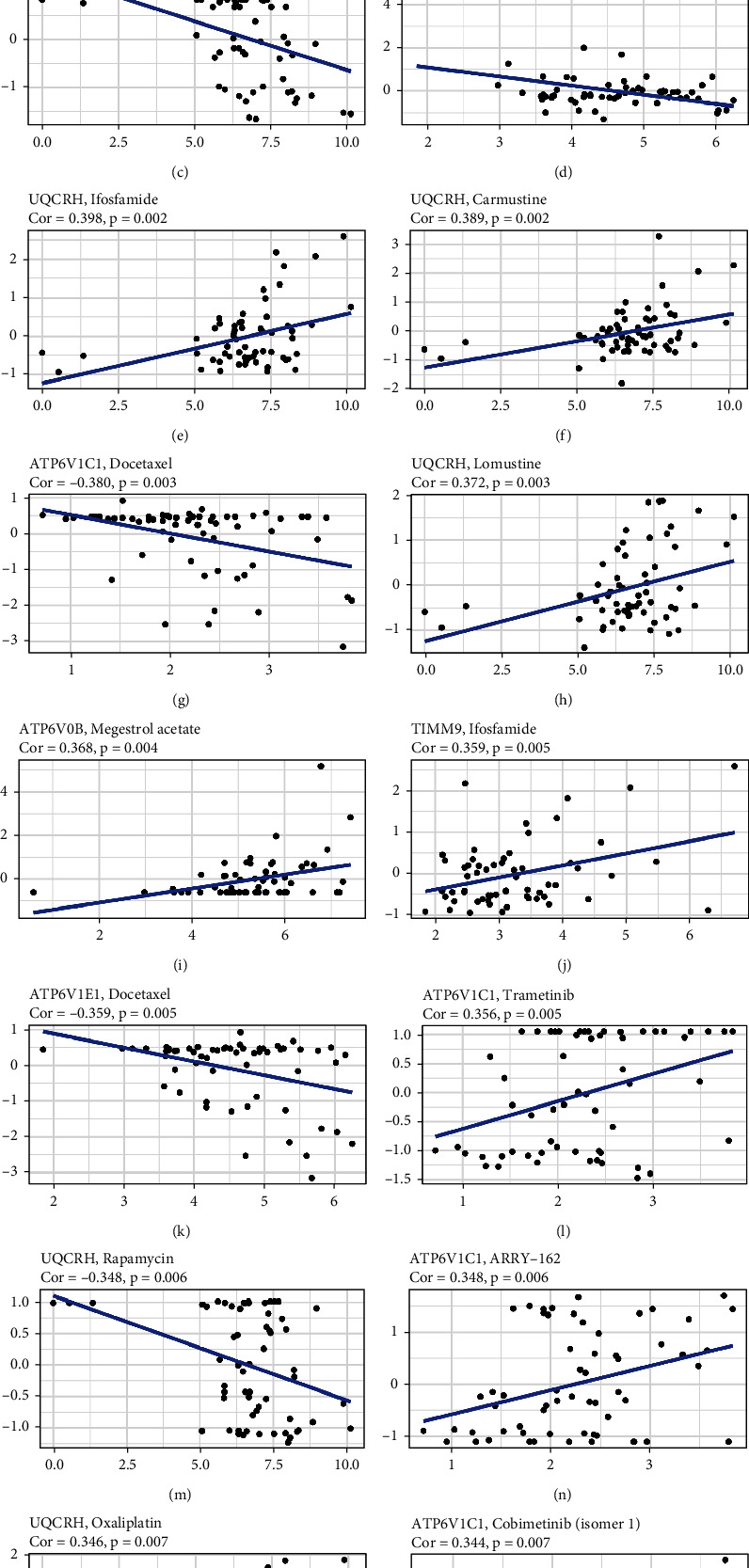
Scatter plots of the top sixteen kinds of associations between the five genes of the prognostic model and chemotherapy drug sensitivity.

We explored the expression of the five signature genes in NCI-60 cell lines and analyzed the associations between their expression levels and drug sensitivity. The results indicated that the five genes were linked with the sensitivity of certain chemotherapy drugs (*P* < 0.05, Figure 8). For example, UQCRH gene expression in tumor cells was positively associated with the sensitivity of asparaginase, ifosfamide, carmustine, lomustine, and oxaliplatin but negatively associated with everolimus and rapamycin. ATP6V1E1 gene expression was negatively associated with the sensitivity of brigatinib, alectinib, and docetaxel. ATP6V1C1 gene expression was positively associated with the sensitivity of trametinib, cobimetinib, and ARRY-162 but negatively associated with everolimus. The expression of TIMM9 was positively associated with the sensitivity of ifosfamide.

## 4. Discussion

The heterogeneity of HCC continues to be a challenge for the treatment and prognostic evaluation of HCC [22]. Many previous studies have concentrated on searching for prognostic models to predict the outcomes of HCC patients, such as models based on ferroptosis-related genes, inflammatory response-related genes, and epithelial-mesenchymal transition-related genes [22–24]. In recent years, OXPHOS has been shown to be upregulated in various cancers, and OXPHOS inhibitors have been revealed as possible therapeutic agents targeting cancer cell metabolism [10, 25]. Additionally, upregulated OXPHOS metabolic activity in cancer cells can promote tumorigenesis [26]. However, the OXPHOS-associated prognostic model for HCC has not been reported.

In the present study, we thoroughly investigated the expression of 200 OAGs in HCC patients, comparing tumor samples to normal samples. Twelve OAGs were identified as candidate genes for model construction. Interestingly, two of the 12 genes, ALDH6A1 and ETFDH, were downregulated in the tumor group, but the other ten genes were upregulated compared with the normal group. Studies have shown that ETFDH and ALDH6A1 might be tumor suppressor genes [27, 28], which might explain this trend. Subsequently, a novel prognostic model involving five OAGs was established by LASSO Cox regression based on the above 12 candidate genes and validated in the other cohort. Similar to the aforementioned prognostic models, this model also had high predictive accuracy for the OS of patients with HCC. In addition, the five-gene model was demonstrated to be an independent prognostic predictor for HCC. The prognostic model was composed of five genes, namely, ATP6V0B, ATP6V1C1, ATP6V1E1, TIMM9, and UQCRH. The expression of the five genes was upregulated in tumor samples and positively correlated with risk scores and hazard ratios, implying that increased OXPHOS activity might be associated with poor prognosis in certain HCC subgroups. Then, we further analyzed the functional enrichment of the DEGs between the low- and high-risk groups. The results revealed that the DEGs were considerably enriched in a series of mitotic processes and cell cycle pathways. Previous studies have indicated that mitosis and proliferation are highly dependent on ATP synthesis, which is mainly produced by the OXPHOS metabolic pathway in the mitochondria [29, 30]. Apart from energy transfer, ATP is also involved in signaling pathways and the biosynthesis of a variety of messengers [31]. Additional studies have identified the roles of ATP in diverse pathological conditions, including inflammation and cancer, and shown that extracellular ATP signaling plays a role in tumor cell proliferation, tumor microenvironment, and immune cell regulation [7, 31]. Interestingly, three genes (ATP6V0B, ATP6V1C1, and ATP6V1E1) in this prognostic model are subunits of vacuolar ATPase (V-ATPase), which hydrolyzes ATP to release energy and transports protons. The emerging roles of V-ATPase include amino acid sensing, glucose sensing, and Wnt and Notch signaling [32]. Besides, certain studies have demonstrated that V-ATPases are conducive to the survival and metastasis of cancer cells through preservation of neutral cytosolic pH [32]. Inhibitors of V-ATPase have been shown to facilitate apoptosis in cancer cells [33]. The present study demonstrated that the three previously mentioned subunits of the V-ATPase were upregulated in HCC tumor samples and inversely correlated with the OS of patients with HCC, except for ATP6V1C1. A recent study demonstrated that impaired OXPHOS limits T cell proliferation in response to antigenic stimulation [34]. Moreover, the V-ATPase appears to be engaged in immune signaling that affects the tumor microenvironment [33]. We then further analyzed the immune cell infiltration, responses, and immune subtypes in the low- and high-risk groups. The results showed that the risk scores were associated with certain innate immune cell infiltrates such as macrophages, aDCs, and NK cells. Moreover, the risk scores were associated with certain immune responses such as T cell co-inhibition and type II IFN response. High-risk scores were associated with wound healing, whereas low-risk scores were associated with the inflammatory pathway. Through tumor microenvironment analysis, we found that the risk scores were positively associated with RNAss but negatively associated with stromal scores. The above results suggest that associations exist between OXPHOS and immune cell regulation as well as the tumor microenvironment, which is consistent with earlier studies. Another study has shown that V-ATPases can facilitate cancer cell survival by preventing cancer drugs from reaching their targets [35]. Thus, we examined the associations between the five genes of the model and the sensitivity of chemotherapy drugs. The results indicated that the expression levels of the five genes were associated with drug sensitivity, especially for the three subunits of ATPase, suggesting that targeting ATPases might be a potential strategy to improve the treatment efficacy for HCC.

TIMM9 is a mitochondrial protein that is expressed in various cancer cells depending on the cancer type. Overexpression of TIMM9 is associated with vascular invasion in gastric cancers and is inversely correlated with the survival of patients with gastric cancer, which indicates that TIMM9 can be used as a marker to predict the outcomes of patients with gastric cancer [36]. In our study, TIMM9 expression was also elevated in HCC tumor samples compared to nontumor liver samples. Moreover, the survival analysis revealed that TIMM9 expression is adversely associated with the OS of patients with HCC. UQCRH is a hinge protein for the mitochondrial electron transport chain that participates in the processes of electron transfer reaction in the OXHPOS pathway. Certain types of cancer are linked with the expression of UQCRH. A study demonstrated that UQCRH expression was positively associated with survival in patients with renal cell carcinoma, suggesting that UQCRH may act as a tumor suppressor gene in renal cancer [37, 38]. However, another study indicated that UQCRH expression was elevated in HCC and that its overexpression is associated with poor prognosis in HCC patients [39]. Additionally, UQCRH can be used as a prognostic predictor for lung adenocarcinoma [40]. The present study also indicated that UQCRH was upregulated in HCC tumor samples and that overexpression of UQCRH was inversely associated with worse OS in patients with HCC. However, the exact mechanisms of TIMM9 and UQCRH involvement in HCC remain unknown.

Our study has three main limitations. First, experimental research is required to corroborate the results discovered by our bioinformatics analysis. Second, this study was designed through retrospective analysis, and prospective studies should be conducted to verify the model's efficiency. Third, the precise mechanisms of the OXPHOS-associated prognostic genes involved in the occurrence and development of HCC remain unknown and need to be explored in our future study.

All in all, this study identified a novel OXPHOS-associated prognostic model with strong predictive capacity. To our knowledge, this is the first OXPHOS-associated gene signature that can predict the prognosis of patients with HCC. This gene signature was significantly associated with mitosis and cell cycle pathway and partly involved in immune cell infiltration, immune functions, TME, and drug sensitivity in HCC. The study provides new understanding of the role of OXPHOS in HCC, which might contribute to individualized treatment and prognosis evaluation.

## Figures and Tables

**Table 1:** Five OXPHOS-associated genes model in the TCGA cohort constructed by LASSO.

Gene name	Univariate cox regression analysis		Differential gene expression analysis		LASSO coefficient
	Hazard ratio	*P* value	LogFc	*P* value	
ATP6V0B	1.6023	0.0002	1.0301	1.13e-18	0.1025
ATP6V1C1	1.4395	0.0036	1.6496	1.00e-24	0.1635
ATP6V1E1	1.7355	0.0019	1.0763	3.47e-25	0.0086
TIMM9	1.8805	0.0003	1.0469	9.02e-23	0.2387
UQCRH	1.6633	5.31 e-05	1.2455	2.01e-22	0.2341

## Data Availability

The datasets used and analyzed during the present study are available from the corresponding author upon rational request.

## Supplementary Materials

Supplementary Materials Supplementary Figure S1. Study scheme of data collection and analysis. Supplementary Figure S2. Prognostic model constructed by LASSO Cox regression. (a) Cross-validation for tuning parameter screening in the LASSO Cox regression model. (b) The coefficient profiles of the LASSO Cox regression model. Supplementary Table S1. OXPHOS-associated genes. Supplementary Table S2. The annotated gene set file applied in ssGSEA. Supplementary Table S3. The chemotherapy drugs involved in the analysis. Supplementary Table S4. The clinical data of the HCC patients involved in this study.
