# Supplementary material for: A Novel Oxidative Phosphorylation-Associated Gene Signature for Prognosis Prediction in Patients with Hepatocellular Carcinoma
Source: Dis Markers. 2022 Sep 5;2022:3594901. doi: 10.1155/2022/3594901 (PMC9467772; doi:10.1155/2022/3594901)
Supplement: Supplementary Materials — Supplementary Figure S1. Study scheme of data collection and analysis. Supplementary Figure S2. Prognostic model constructed by LASSO Cox regression. (a) Cross-validation for tuning parameter screening in the LASSO Cox regression model. (b) The coefficient profiles of the LASSO Cox regression model. Supplementary Table S1. OXPHOS-associated genes. Supplementary Table S2. The annotated gene set file applied in ssGSEA. Supplementary Table S3. The chemotherapy drugs involved in the analysis. Supplementary Table S4. The clinical data of the HCC patients involved in this study. [file 3594901.f1.pdf]

Supplementary Figure S1

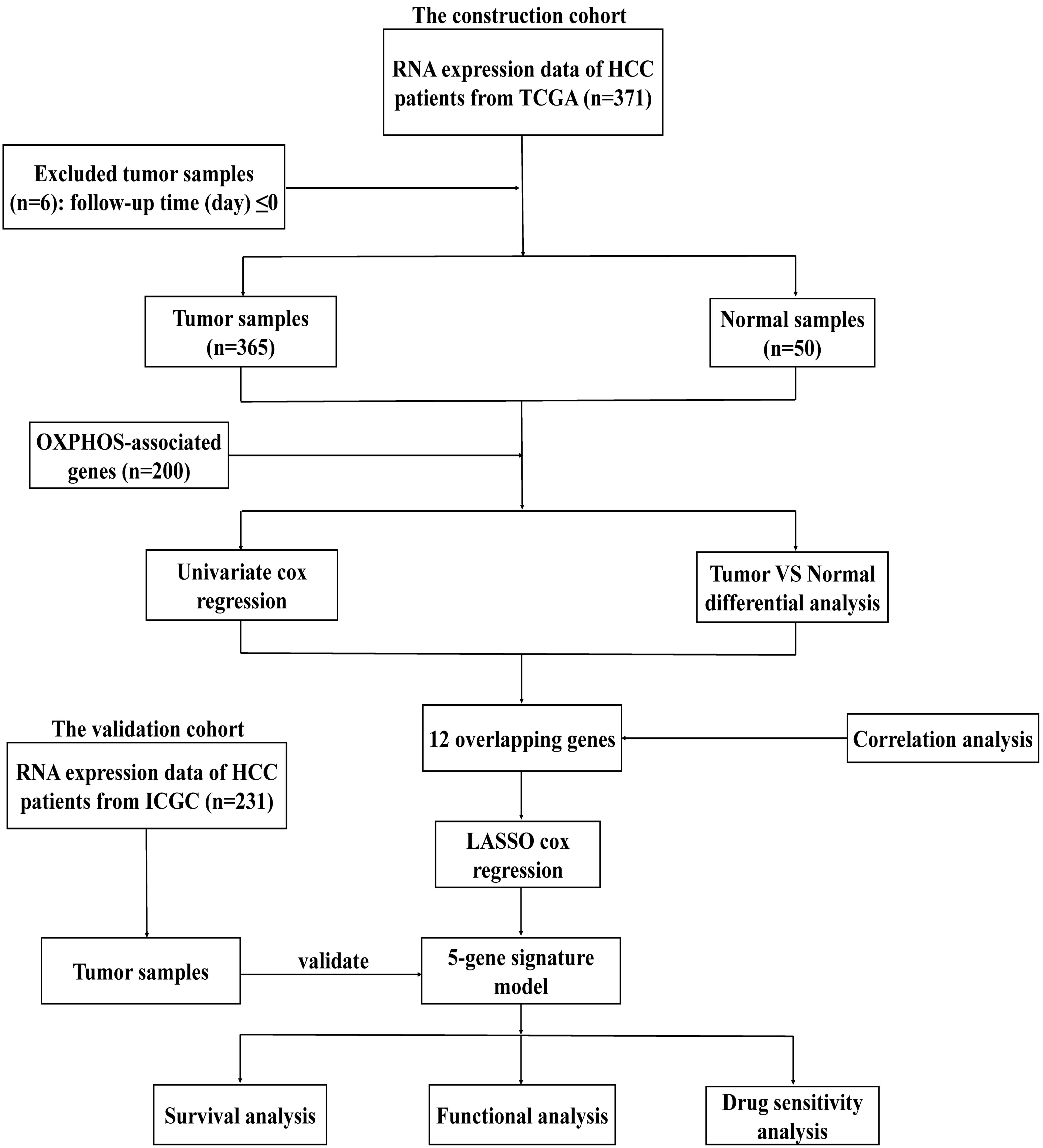

Suppplementary Figure S2

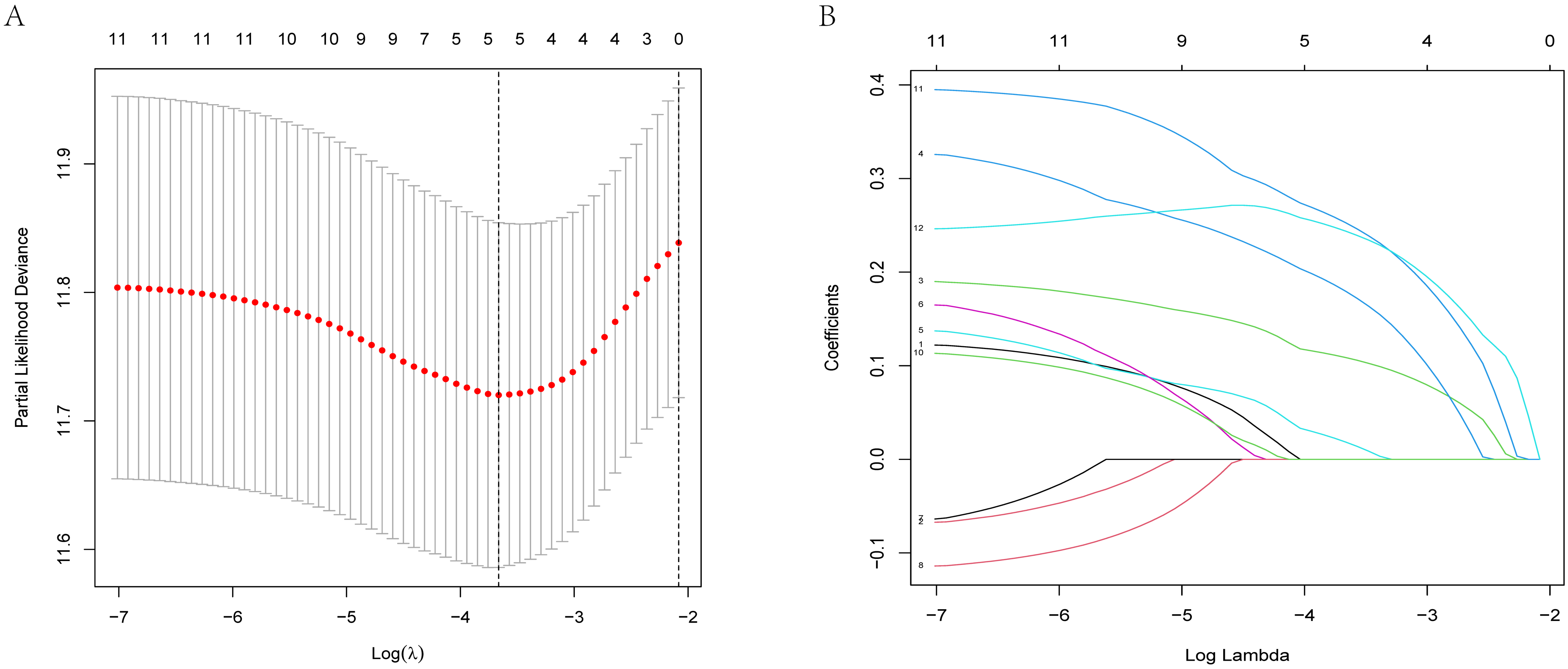

Supplementary Table S1. OXPHOS-associated genes

Gene name						
ABCB7	BDH2	GRPEL1	NDUFA5	POR	ATP6V0B	MRPS12
ACAA1	CASP7	HADHA	NDUFA6	PRDX3	ATP6V0C	MRPS15
ACAA2	COX10	HADHB	NDUFA7	RETSAT	ATP6V0E1	MRPS22
ACADM	COX11	HCCS	NDUFA8	RHOT1	ATP6V1C1	MRPS30
ACADSB	COX15	HSD17B10	NDUFA9	RHOT2	ATP6V1D	MTRF1
ACADVL	COX17	HSPA9	NDUFAB1	SDHA	ATP6V1E1	MTRR
ACAT1	COX4I1	HTRA2	NDUFB1	SDHB	ATP6V1F	MTX2
ACO2	COX5A	IDH1	NDUFB2	SDHC	ATP6V1G1	NDUFA1
AFG3L2	COX5B	IDH2	NDUFB3	SDHD	ATP6V1H	NDUFA2
AIFM1	COX6A1	IDH3A	NDUFB4	SLC25A11	BAX	NDUFA3
ALAS1	COX6B1	IDH3B	NDUFB5	SLC25A12	BCKDHA	NDUFA4
ALDH6A1	COX6C	IDH3G	NDUFB6	SLC25A20	ECI1	OPA1
ATP1B1	COX7A2	IMMT	NDUFB7	SLC25A3	ETFA	OXA1L
ATP5F1A	COX7A2L	ISCA1	NDUFB8	SLC25A4	ETFB	PDHA1
ATP5F1B	COX7B	ISCU	NDUFC1	SLC25A5	ETFDH	PDHB
ATP5F1C	COX7C	LDHA	NDUFC2	SLC25A6	FDX1	PDHX
ATP5F1D	COX8A	LDHB	NDUFS1	SUCLA2	FH	PDK4
ATP5F1E	CPT1A	LRPPRC	NDUFS2	SUCLG1	FXN	PDP1
ATP5MC1	CS	MAOB	NDUFS3	SUPV3L1	GLUD1	PHB2
ATP5MC2	CYB5A	MDH1	NDUFS4	SURF1	GOT2	PHYH
ATP5MC3	CYB5R3	MDH2	NDUFS6	TCIRG1	GPI	PMPCA
ATP5ME	CYC1	MFN2	NDUFS7	TIMM10	GPX4	POLR2F
ATP5MF	CYCS	MGST3	NDUFS8	TIMM13	UQCR10	UQCRH
ATP5MG	DECR1	MPC1	NDUFV1	TIMM17A	UQCR11	UQCRQ
ATP5PB	DLAT	MRPL11	NDUFV2	TIMM50	UQCRB	VDAC1
ATP5PD	DLD	MRPL15	NNT	TIMM8B	UQCRC1	VDAC2
ATP5PF	DLST	MRPL34	NQO2	TIMM9	UQCRC2	
ATP5PO	ECH1	MRPL35	OAT	TOMM22	UQCRFS1	
ATP6AP1	ECHS1	MRPS11	OGDH	TOMM70	VDAC3	

Supplementary Table S2. The annotated gene set file applied in ssGSEA

Immune types	Genes
aDCs	CD83 LAMP3 CCL1
APC_co_inhibition	C10orf54 CD274 LGALS9 PDCD1LG2 PVRL3
APC_co_stimulation	CD40 CD58 CD70 ICOSLG SLAMF1 TNFSF14 TNFSF15 TNFSF18 TNFSF4 TNFSF8 TNFSF9
B_cells	BACH2 BANK1 BLK BTLA CD79A CD79B FCRL1 FCRL3 HVCN1 RALGPS2
CCR	CCL16 TPO TGFBR2 CXCL2 CCL14 TGFBR3 IL11RA CCL11 IL4I1 IL33 CXCL12 CXCL10 BMPER BMP8A CXCL11 IL21R IL17B TNFRSF9 ILF2 CX3CR1 CCR8 TNFSF12 CSF3 TNFSF4 BMP3 CX3CL1 BMP5 CXCR2 TNFRSF10D BMP2 CXCL14 CCL28 CXCL3 BMP6 CCL21 CXCL9 CCL23 IL6 TNFRSF18 IL17RD IL17D IL27 CCL7 IL1R1 CXCR4 CXCR2P1TGFB1I1 IFNGR1 IL9R IL1RAPL1IL11 CSF1 IL20RA IL25 TNFRSF4 IL18 ILF3 CCL20 TNFRSF12A IL6ST CXCL13 IL12B TNFRSF8 IL6R BMPR2 IFNEIL1RAPL2IL3RA BMP4 CCL24 TNFSF13B CCR4 IL2RA IL32 TNFRSF10C IL22RA1 BMPR1A CXCR5 CXCR3 IFNA8 IL17REL IFNB1 IFNAR1 TNFRSF1B CCL17 IFNL1 IL16 IL1RL1 ILK CCL25 ILDR2 CXCR1 IL36RN IL34 TGFB1 IFNG IL19 ILKAP BMP2K CCR10 ILDR1 EPO CCR7 IL17C IL23A CCR5 IL7 EPOR CCL13 IL2RG IL31RA TNFAIP6 IFNL2 BMP1 IL12RB1 TNFAIP8 IL4R TNFRSF6B TNFAIP8L1 TNFRSF10B IFNL3 CCL5 CXCL6 CXCL1 CCR3 TNFSF11 CSF1R IL21 IL1RAP IL12RB2 CCL1 IL17RA CCR1 IL1RN TNFRSF11B TNFRSF14 IL13 IL2RB BMP8B CCL2 IL24 IL18RAP TGFBI TNFSF10 TNFRSF11A CXCL5 IL5RA TNFSF9 IL1RL2 TNFRSF13C IL36G IL15RA TNFRSF21 CXCL8 IL22RA2 TNFAIP8L2 IL18R1 IFNLR1 CXCR6 CCL3L3 TNFRSF1A IL17RE IFNGR2 IL17RC TNFAIP8L3 ILVBL TGFBRAP1 CCL4L1 CSF2RA CCRN4L CCL26 TNFAIP1 CCRL2 IFNA10 TNFRSF17 IFNA13 IL20 IL18BP CCL3L1 TNFSF12-TNFSF13 IL5 IL23R IL26 TNF TGFA CSF2 IL1F10 CXCL17 TNFSF13 IFNA4 IL37 IL12A IL7R IFNA1 IL1A IL4 IL2 CCL22 CSF3R IL10 IFNK TGFB2 IL1R2 IL1B IL17F IL27RA IL15 TNFSF8 IL36B XCL1 CXCL16 TNFRSF19 IL3 CCL3 IFNA2 BMPR1B IFNA21 TNFSF18 CCL8 IL17RB TNFRSF25 IL22 IL10RB IFNAR2 CCL18 IFNA16 CSF2RB IL36A TNFAIP3 IL13RA2 IL13RA1 CCR9 TNFRSF10A IFNA7 IFNW1 XCL2 TNFSF14 CCR2

	BMP15 BMP10 CCL15-CCL14 TGFBR1 IFNA5 BMP7 IFNA14 IL20RB IL10RA IFNA17 CCR6 TGFB3 CCL15 CCL4 CCL27 TNFRSF13B TNFAIP2 IL31 IL17A TNFSF15 CCL19 IFNA6 IL9
CD8+_T_cells	CD8A
Check-point	IDO1 LAG3 CTLA4 TNFRSF9 ICOS CD80 PDCD1LG2 TIGIT CD70 TNFSF9 ICOSLG KIR3DL1 CD86 PDCD1 LAIR1 TNFRSF8 TNFSF15 TNFRSF14 IDO2 CD276 CD40 TNFRSF4 TNFSF14 HHLA2 CD244 CD274 HAVCR2 CD27 BTLA LGALS9 TMIGD2 CD28 CD48 TNFRSF25 CD40LG ADORA2A VTCN1 CD160 CD44 TNFSF18 TNFRSF18 BTNL2 C10orf54 CD200R1 TNFSF4 CD200 NRP1
Cytolytic_activity	PRF1 GZMA
DCs	CCL22 CD209 CCL13 CCL17
HLA	HLA-E HLA-DPB2 HLA-C HLA-J HLA-DQB1 HLA-DQB2 HLA-DQA2 HLA-DQA1 HLA-A HLA-DMA HLA-DOB HLA-DRB1 HLA-H HLA-B HLA-DRB5 HLA-DOA HLA-DPB1 HLA-DRA HLA-DRB6 HLA-L HLA-F HLA-G HLA-DMB HLA-DPA1
iDCs	CD1A CD1E
Inflammation-promoting	CCL5 CD19 CD8B CXCL10 CXCL13 CXCL9 GNLY GZMB IFNG IL12A IL12B IRF1 PRF1 STAT1 TBX21
Macrophages	C11orf45 CD68 CLEC5A CYBB FUCA1 GPNMB HS3ST2 LGMN MMP9 TM4SF19
Mast_cells	CMA1 MS4A2 TPSAB1
MHC_class_I	B2M HLA-A TAP1
Neutrophils	EVI2B HSD17B11 KDM6B MEGF9 MNDA NLRP12 PADI4 SELL TRANK1 VNN3
NK_cells	KLRC1 KLRF1
Parainflammation	CXCL10 PLAT CCND1 LGMN PLAUR AIM2 MMP7 ICAM1 MX2 CXCL9 ANXA1 TLR2 PLA2G2D ITGA2 MX1 HMOX1 CD276 TIRAP IL33 PTGES TNFRSF12A SCARB1 CD14 BLNK IFIT3 RETNLB IFIT2 ISG15 OAS2 REL OAS3 CD44 PPARG BST2 OAS1 NOX1 PLA2G2A IFIT1 IFITM3 IL1RN
pDCs	CLEC4C CXCR3 GZMB IL3RA IRF7 IRF8 LILRA4 PHEX PLD4 PTCRA
T_cell_co-inhibition	BTLA C10orf54 CD160 CD244 CD274 CTLA4 HAVCR2 LAG3 LAIR1 TIGIT
T_cell_co-stimulation	CD2 CD226 CD27 CD28 CD40LG ICOS SLAMF1 TNFRSF18 TNFRSF25 TNFRSF4 TNFRSF8 TNFRSF9 TNFSF14

T_helper_cells	CD4
Tfh	PDCD1 CXCL13 CXCR5
Th1_cells	IFNG TBX21 CTLA4 STAT4 CD38 IL12RB2 LTA CSF2
Th2_cells	PMCH LAIR2 SMAD2 CXCR6 GATA3 IL26
TIL	ITM2C CD38 THEMIS2 GLYR1 ICOS F5 TIGIT KLRD1 IRF4 PRKCQ FCRL5 SIRPG LPXN IL2RG CCL5 LCK TRAF3IP3 CD86 MALLILRB1 DOK2 CD6 PAG1 LAX1 PLEK PIK3CD SLAMF1 XCL1 GPR171 XCL2 TBX21 CD2 CD53 KLHL6 SLAMF6 CD40 SIT1 TNFRSF4 CD79A CD247 LCP2 CD3D CD27 SH2D1A FYB ARHGAP30 ACAP1 CST7 CD3G IL2RB CD3E FCRL3 CORO1A ITK TCL1A CYBB CSF2RB IKZF1 NCF4 DOCK2 CCR2 PTPRC PLAC8 NCKAP1L IL7R 6-Sep CD28 STAT4 CD8A LY9 CD48 HCST PTPRCAP SASH3 ARHGAP25 LAT TRAT1 IL10RA PAX5 CCR7 DOCK11 PARVG SPNS1 CD52 HCLS1 ARHGAP9 GIMAP6 PRKCB MS4A1 GPR18 TBC1D10C GVINP1 P2RY8 EVI2B VAMP5 KLRK1 SELLMPEG1 MS4A6A ARHGAP15 MFNG GZMK SELPLG TARP GIMAP7 FAM65B INPP5D ITGA4 MZB1 GPSM3 STK10 CLEC2D IL16 NLRC3 GIMAP5 GIMAP4 IFFO1 CFH PVRIG CFHR1
Treg	IL12RB2 TMPRSS6 CTSC LAPTM4B TFRC RNF145 NETO2 ADAT2 CHST2 CTLA4 NFE2L3 LIMA1 IL1R2 ICOS HSDL2 HTATIP2 FKBP1A TIGIT CCR8 LTA SLC35F2 IL21R AHCYL1 SOCS2 ETV7 BCL2L1 RRAGB ACSL4 CHRNA6 BATF LAX1 ADPRH TNFRSF4 ANKRD10 CD274 CASP1 LY75 NPTN SSTR3 GRSF1 CSF2RB TMEM184C NDFIP2 ZBTB38 ERI1 TRAF3 NAB1 HS3ST3B1 LAYN JAK1 VDR LEPROT GCNT1 PTPRJ IKZF2 CSF1 ENTPD1 TNFRSF18 METTL7A KSR1 SSH1 CADM1 IL1R1 ACP5 CHST7 THADA CD177 NFAT5 ZNF282 MAGEH1
Type_I_IFN_Reponse	DDX4 IFIT1 IFIT2 IFIT3 IRF7 ISG20 MX1MX2 RSAD2 TNFSF10
Type_II_IFN_Reponse	GPR146 SELP AHR

Supplementary Table S3. The chemotherapy drugs involved in the analysis

Drug name			
METHOTREXATE	Megestrol acetate	Homoharringtonine	7-Ethyl-10-hydroxycamptothecin
6-THIOGUANINE	tfdu	Mithramycin	Bortezomib
6-MERCAPTOPURINE	Procarbazine	Tegafur	Irofulven
Nitrogen mustard	Lomustine	Parthenolide	Temsirolimus
Allopurinol	Daunorubicin	Dexrazoxane	Denileukin Diftitox Ontak
Actinomycin D	Daunorubicin	Tamoxifen	Pemetrexed
Chlorambucil	STREPTOZOCIN	PENTOSTATIN	Vorinostat
Thiotepa	Calusterone	RAPAMYCIN	Estramustine
Melphalan	Estramustine	Carboplatin	Arsenic trioxide
Triethylenemelamine	Vinblastine	Valrubicin	Eribulin mesilate
Dromostanolone Propionate	Fluphenazine	Idarubicin	Gefitinib
Acrichine	Arsenic trioxide	Epirubicin	Erlotinib
Fluorouracil	AZACITIDINE	Oxaliplatin	Fulvestrant
Nandrolone phenpropionate	Cladribine	MITOXANTRONE	Celecoxib
TESTOLACTONE	Mithramycin	Cytarabine	Zoledronate
Mithramycin	Asparaginase	Mitoxantrone	Belinostat
Pipobroman	Ifosfamide	Fludarabine	Lapatinib
Cyclophosphamide	Acetalax	Imiquimod	Irinotecan
Mitomycin	Fludarabine	Carmustine	Dasatinib
Floxuridine	Cisplatin	Mithramycin	Everolimus
Hydroxyurea	Isotretinoin	Rapamycin	Pazopanib
Uracil mustard	Teniposide	Clofarabine	Selumetinib
Dexamethasone Decadron	Doxorubicin	Vinorelbine	Imatinib
Mitotane	Fludarabine	Topotecan	Lapatinib
DACARBAZINE	Bleomycin	Gemcitabine	Nelfinavir
Vinblastine	Paclitaxel	Bisacodyl, active ingredient of Viraplex	Nilotinib
Acetalax	DECITABINE	Irinotecan	Olaparib
Cytarabine	Mitomycin	Docetaxel	Ixabepilone

Vincristine	Bendamustine	Depsipeptide	Raloxifene
Raltitrexed	Etoposide	Simvastatin	Midostaurin

Supplementary Table S4.The clinical data of the HCC patients involved in this study

HCC patients	TCGA cohort (n=365)	ICGC cohort (n=231)
**Age(median,range) (years)**	57(16-90)	67(31-89)
**Gender**		
Male	246	170
Female	119	61
**Grade**		
1	55	na
2	175	na
3	118	na
4	12	na
unknown	5	na
**Stage**		
I	170	36
II	84	105
III	83	71
IV	4	19
unknown	24	0
**Satus**		
Alive	235	189
Dead	130	42
